# Head and neck melanoma: outcome and predictors in a population-based cohort study

**DOI:** 10.1186/s13005-021-00295-x

**Published:** 2021-10-22

**Authors:** Steffen Spoerl, Gerrit Spanier, Elena Reiter, Michael Gerken, Sebastian Haferkamp, Jirka Grosse, Konstantin Drexler, Tobias Ettl, Monika Klinkhammer-Schalke, René Fischer, Silvia Spoerl, Torsten E. Reichert, Christoph Klingelhöffer

**Affiliations:** 1 Department of Cranio-Maxillofacial Surgery, University Hospital Regensburg, D-93042 Regensburg, Germany; 2 Tumor Center - Institute for Quality Management and Health Services Research, University of Regensburg, Regensburg, Germany; 3 Department of Dermatology, University Hospital Regensburg, Regensburg, Germany; 4 Department of Nuclear Medicine, University Hospital Regensburg, Regensburg, Germany; 5 Department of Otorhinolaryngology, University Hospital Regensburg, Regensburg, Germany; 6 Department of Internal Medicine 5 - Hematology/Oncology, Friedrich-Alexander University Erlangen-Nürnberg, Erlangen, Germany

**Keywords:** Melanoma, Head and neck, Sentinel lymph node, SLNB, Comorbidities, CCI, Survival, Recurrence

## Abstract

**Background:**

To evaluate predictive clinico-pathological characteristics on outcome in head and neck melanoma (HNM) in a population-based study with particular emphasis on the prognostic effect of sentinel lymph node biopsy (SLNB), Charlson comorbidity index (CCI) and distinct tumor localisations.

**Methods:**

Here we primarily describe a retrospective multicenter population-based cohort study with 402 patients having undergone resection with curative intent of HNM between 2010 and 2017. SLNB was used in the diagnosis of 79 HNM patients. Outcome was analyzed, focusing on SLNB, CCI as well as tumor localisation. Overall survival (OAS) und recurrence free survival (RFS) was examined by uni- and multivariate analysis.

**Results:**

Histopathologically verified lymph node metastasis according to SLNB was associated with impaired RFS in HNM patients (*p* = 0.004). Especially in higher tumor stages, the sole implementation of SLNB improved survival significantly in the present cohort (*p* = 0.042). With most of the HNM being located in the face, melanoma of the scalp and neck could be linked to deteriorated patient’s outcome in uni- as well as multivariate analysis (*p* = 0.021, *p* = 0.004).

**Conclusions:**

SLNB is a useful tool in predicting development of distant metastasis after HNM resection with curative intent. Especially in higher tumor stages, performing a SLNB ameliorated survival of HNM patients. Additionally, CCI as well as a distinct tumor localisations in HNM were identified as important risk factors in our population-based cohort study.

## Background

With an incidence of 300,000 cases worldwide, the head and neck region is one of the most common tumor sites for melanoma [1, 2]. Particularly in head and neck melanoma (HNM), a growing proportion of patients is diagnosed with quite early tumor stages accompanied with almost unchanged life expectancy [3]. However, with increasing thickness of HNM, the probability of occult regional metastasis rises [4].

A promising tool limiting adverse prognosis due to occult regional metastasis was primarily described by Morton et al.. Here especially for earlier stages of HNM patients, alternative therapies to perform an elective neck dissection (END), which often results in postoperative adverse events, were searched. In this context, sentinel lymph node biopsy (SLNB) successfully identified sentinel lymph nodes in individual drainage pathways with a false negative rate of under 1% and a reduced donor site morbidity [5]. In the MSLT trial, an international multicenter trial being initiated in 1994, the role of clinically and radiologically based nodal observation after resection with curative intent or concomitant SLNB with primary tumor resection was evaluated in intermediate thickness melanoma. In this regard, SLNB was identified as a powerful staging tool with a profound prognostic value for survival of melanoma patients [6].

Nevertheless, SLNB in the head and neck raises several difficulties, mainly through variable lymphatic drainage patterns, of which 34% were diagnosed not in agreement with earlier clinical prediction [7, 8]. These conventionally not predictable lymphatic areas are of unclear biological or clinical relevance, because few of the incongruous sites actually contain metastatic disease. Despite this, they can still harbor melanoma. Accordingly, tumor localisations marked on preoperative lymphoscintigraphy need to be surgically explored [9]. A further challenging aspect is the anatomical complexity and density of functional structures, vessels and nerves in the head and neck region. With the surgical approach for SLNB (and of course HNM resection and reconstruction) one needs to preserve relevant anatomy, which otherwise could lead to unfavorable functional and esthetic sequelae [10].

One major objective of this population-based multicenter cohort study was to evaluate the prognostic significance of SLNB on survival and disease recurrence in different tumor stages of HNM patients. Additionally, further prognostic parameters were included in this study, aiming to stratify risk factors and indicating the individual necessity for adjuvant therapies.

## Methods

### Patient selection

In this population-based multicenter cohort study, we analyzed data of primarily resected HNM patients using the database of the Clinical Cancer Registry at the Tumor Center Regensburg in Eastern Bavaria. This region of Germany covers a population of around 2.3 million inhabitants. All patients had been examined and treated for a newly diagnosed HNM between 01/01/2010 and 31/12/2017. All participants have been treated at the Departments of Cranio-Maxillofacial Surgery, Dermatology and Otorhinolaryngology at University Hospital Regensburg and a presentation at a multidisciplinary tumor board was mandatory. Patients with previous HNM, *non in sano* resection, incomplete tumor characteristics or staging information as well as patients with neoadjuvant treatment were excluded. Staging was performed according to the 7th edition of the American Joint Committee on Cancer (AJCC) cancer staging and manual [11]. Clinical and histological patient data were retrieved from written and electronical medical records. Charlson Comorbidity Index (CCI) was calculated as previously described without taking HNM into account [12]. Adjuvant treatment was based on the recommendation of the multidisciplinary tumor board and chemo- and/or immunotherapy was used accordingly.

Disease relapse was defined as local disease recurrence or distant metastasis by radiologic evidence with clinical correlation or histologic confirmation with biopsy. Recurrence free survival (RFS) and overall survival (OAS) was calculated based on follow-up data from medical records, death certificates, registration offices, and the Clinical Cancer Registry. Mean follow-up was 4.5 years (median 4.7 years).

### Sentinel lymph node scintigraphy

SLN scintigraphy was carried out according to the practical guidelines for lymphoscintigraphy and SLNB in melanoma of the European Association of Nuclear Medicine [13] and the german S3-guidline of melanoma treatment [14]. Hereby, a SLNB was recommended if tumor thickness was larger than 1 mm or additional risk factors like ulceration or an increased rate of mitosis were present. The implementation of SLNB was achieved by a defined protocol. The day prior to surgery, four intradermal peritumoral injections with 20 MBq ^99m^Tc-labeled human serum albumin colloid (Nanocoll®, GE Healthcare, Chicago, IL, USA) were applied. After imaging, SLNB was carried out using a portable gamma probe. Consecutively, all dissected lymph nodes were analyzed histopathologically. A false negative SLNB was assigned, if regional recurrence was detected in the exact lymphatic drainage of the previously performed SLNB.

### Statistics

Metric variables were analyzed for differences in their means using student’s *t-*test in case of log-normal distribution, otherwise using Mann-Whitney U-test. Independence of categorical variables was analyzed using Pearson’s chi-squared test. OAS, RFS and cumulative recurrence rates were calculated from date of resection to date of death, date of first recurrence or date last alive until cut-off date 30/06/2019, using the Kaplan-Meier and Cox regression method. Differences in outcome estimates were tested using the log-rank-test. For risk adjustment, multivariate Cox regression was applied. Results were reported with hazard ratios (HRs) and 95% confidence intervals (CIs). A *p*-value < 0.05 was considered significant for all tests. All analyses were performed using IBM SPSS Statistics Version 25.0 (IBM Corp., Armonk, N.Y., USA).

## Results

**Fig. 1:**
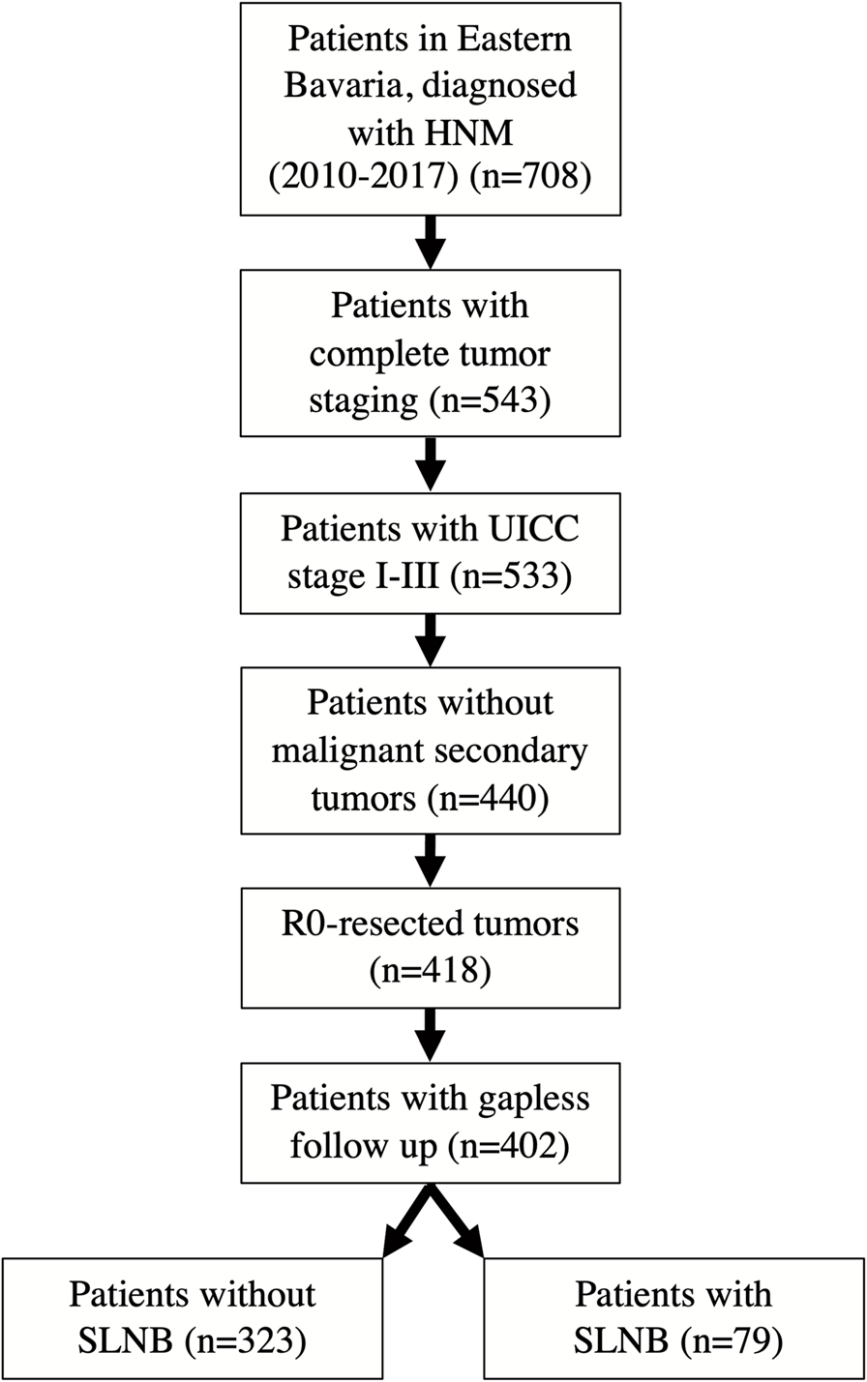
STARD (Standards for Reporting of Diagnostic Accuracy Studies) flow diagram

For the entire cohort (*n* = 402), inclusion criteria are illustrated in Fig. 1, clinico-pathological characteristics are summarized in Tab. 1. In total 47.4% of patients were female, mean age was 65.2 years with a mean age at diagnosis of 63.9 years for men and 68.0 years for women. Most common tumor localisation was the face (67.7%), the other 32.3% were diagnosed on the scalp and neck. Union for International Cancer Control (UICC) stage IA could be attributed to 54.5% of patients, whereas 91 patients (22.6%) were staged UICC class II and 20 patients (5.0%) were assigned to UICC class III. Histopathological subgroups were evaluated by using the paraffin-embedded tissue, which consecutively received staining with haematoxylin and eosin. Histopathological workup hereby revealed superficial spreading melanoma (31.3%) and lentigo maligna melanoma (41.3%) as the most common types. In 76.4% of patients, tumor resection margins were ≥ 5 mm, adjuvant chemo−/immunotherapy was administered to 18 patients (4.5%).

**Table 1:** Patients clinico-pathological characteristics according to SLNB (*n* = 402)

		SLNB						χ2
		no		yes		total		*p*
		Count	%	Count	%	Count	%	
Sex	Male	170	52.6%	57	72.2%	227	56.5%	**0.002**
	Female	153	47.4%	22	27.8%	175	43.5%	
Age at diagnosis	< 50.0	51	15.8%	21	26.6%	72	17.9%	**0.006**
	50.0–59.9	47	14.6%	12	15.2%	59	14.7%	
	60.0–69.9	68	21.1%	12	15.2%	80	19.9%	
	70.0–79.9	95	29.4%	30	38.0%	125	31.1%	
	≥ 80.0	62	19.2%	4	5.1%	66	16.4%	
CCI	0	239	74.0%	57	72.2%	296	73.6%	0.530
	1	57	17.6%	16	20.3%	73	18.2%	
	2	19	5.9%	3	3.8%	22	5.5%	
	3	4	1.2%	3	3.8%	7	1.7%	
	4	3	0.9%	0	0.0%	3	0.7%	
	5	1	0.3%	0	0.0%	1	0.2%	
CCI classified	0	239	74.0%	57	72.2%	296	73.6%	0.739
	≥ 1	84	26.0%	22	27.8%	106	26.4%	
Tumor localisation (ICD-10)	Lip skin (C44.0)	2	0.6%	0	0.0%	2	0.5%	0.062
	Eyelid (C44.1)	16	5.0%	2	2.5%	18	4.5%	
	Outer ear (C44.2)	44	13.6%	17	21.5%	61	15.2%	
	Face (C44.3)	163	50.5%	28	35.4%	191	47.5%	
	Scalp and neck (C44.4)	98	30.3%	32	40.5%	130	32.3%	
Tumor localisation	Face	225	69.7%	47	59.5%	272	67.7%	0.083
	Scalp and neck	98	30.3%	32	40.5%	130	32.3%	
UICC stage	IA	214	66.3%	5	6.3%	219	54.5%	**< 0.001**
	IB	51	15.8%	21	26.6%	72	17.9%	
	II	48	14.9%	43	54.4%	91	22.6%	
	III	10	3.1%	10	12.7%	20	5.0%	
Histological subgroups	Lentigo maligna melanoma	156	48.3%	10	12.7%	166	41.3%	**< 0.001**
	Nodular melanoma	34	10.5%	27	34.2%	61	15.2%	
	Superficial spreading melanoma	104	32.2%	22	27.8%	126	31.3%	
	Melanoma not otherwise specified	22	6.8%	11	13.9%	33	8.2%	
	Other	7	2.2%	9	11.4%	16	4.0%	
Tumor thickness (mm)	< 1	232	71.8%	11	13.9%	243	60.4%	**< 0.001**
	1–2	39	12.1%	26	32.9%	65	16.2%	
	2–4	28	8.7%	16	20.3%	44	10.9%	
	> 4	24	7.4%	26	32.9%	50	12.4%	
Resection margin (mm)	< 5	83	25.7%	12	15.2%	95	23.6%	**< 0.001**
	5–9	137	42.4%	18	22.8%	155	38.6%	
	≥ 10	103	31.9%	49	62.0%	152	37.8%	
Adjuvant Chemo−/ Immunotherapy	yes	12	3.7%	6	7.6%	18	4.5%	0.135
	no	311	96.3%	73	92.4%	384	95.5%	
	total	323	100.0%	79	100.0%	402	100.0%	

**Fig. 2:**
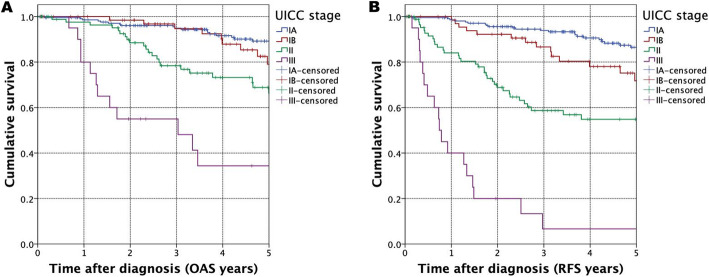
Survival in HNM patients: Kaplan-Meier curves for OAS (Fig. 2A) and RFS (Fig. 2B) for UICC stages with UICC IA set as reference (*p* = 0.132 for UICC IB, *p* < 0.001 for UICC II, *p* < 0.001 for UICC III) and survival curves for RFS (Fig. 2B) with UICC IA set as reference (*p* = 0.008 for UICC IB, *p* < 0.001 for UICC II, *p* < 0.001 for UICC III)

The complete cohort five-year OAS was 79.6% and five-year RFS, was 73.0% respectively. Survival rates for different UICC stages were calculated, resulting in a five-year OAS for UICC stage IA of 89.2, 79.1% for stage IB, 66.3% for stage II and for UICC stage III 34.4% (Fig. 2A, *p* = 0.132 for UICC IB, *p* <  0.001 for UICC II, *p* <  0.001 for UICC III with UICC IA set as reference). Figure 2B shows corresponding survival curves for RFS (*p* = 0.008 for UICC IB, *p* <  0.001 for UICC II, *p* <  0.001 for UICC III). Additionally, tumor stage was correlated with OAS by using multivariate Cox regression with impaired survival for higher UICC stages (II + III) vs. UICC stage I (HR = 3.752, 95% CI = 2.222–6.338, *p* <  0,001) (Tab. 2).

**Table 2:** Survival analysis of RFS for HNM patients according to accomplished SLNB and its results (*n* = 79). RFS was analyzed by using univariate and multivariate Cox regression

		Univariate Cox regression				Multivariate Cox regression			
		*p*	HR	Lower 95%-CI	Upper 95%-CI	*p*	HR	Lower 95%-CI	Upper 95%-CI
SLNB result	No biopsy		1.000				1.000		
	Positive SLN	**< 0.001**	14.442	6.974	29.906	**0.001**	4.046	1.779	9.200
	Negative SLN	0.312	1.333	0.763	2.328	0.311	0.717	0.377	1.365
Age at diagnosis	< 50		1.000				1.000		
	50.0–59.9	**0.040**	2.794	1.047	7.453	**0.002**	5.007	1.814	13.882
	60.0–69.9	**0.021**	2.971	1.178	7.493	**0.001**	5.090	1.963	13.196
	70.0–79.9	**< 0.001**	4.838	2.043	11.460	**< 0.001**	5.774	2.325	14.336
	≥ 80.0	**< 0.001**	7.343	3.030	17.794	**< 0.001**	8.875	3.447	22.853
Sex	Male		1.000				1.000		
	Female	0.136	0.738	0.495	1.101	0.770	1.066	0.694	1.637
UICC stage	I		1.000				1.000		
	II + III	**< 0.001**	5.501	3.710	8.157	**< 0.001**	3.752	2.222	6.338
Localisation	Scalp and neck		1.000				1.000		
	Face	**0.021**	0.625	0.419	0.931	**0.004**	0.498	0.309	0.801
CCI classified	0		1.000				1.000		
	≥ 1	**< 0.001**	2.505	1.685	3.725	0.269	1.291	0.821	2.030
Resection margin (mm)	< 5		1.000				1.000		
	5–9	**0.048**	0.611	0.375	0.995	**0.017**	0.532	0.317	0.893
	≥ 10	0.437	0.826	0.511	1.336	**< 0.001**	0.255	0.145	0.450
Histological subgroups	Lentigo maligna melanoma		1.000				1.000		
	Nodular melanoma	**< 0.001**	5.443	3.339	8.872	**< 0.001**	4.181	2.220	7.876
	Superficial spreading melanoma	0.426	0.792	0.445	1.407	0.885	1.047	0.559	1.964
	Melanoma not otherwise specified	**< 0.001**	3.063	1.703	5.512	**< 0.001**	4.929	2.360	10.293

**Fig. 3:**
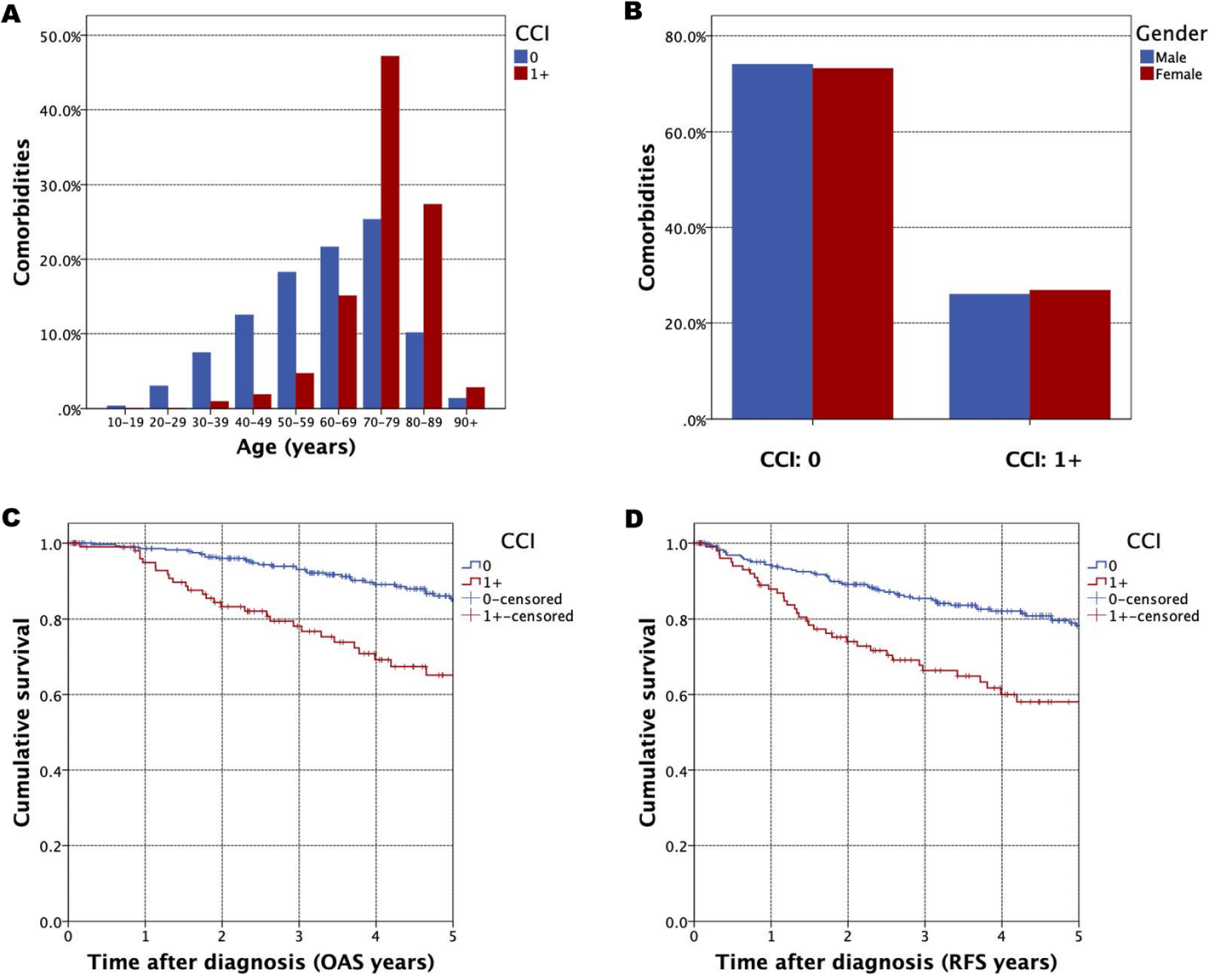
Comorbidities in HNM patients: CCI according to patients age (Fig. 3A) and gender (Fig. 3B); Kaplan-Meier curves for OAS (Fig. 3C) and RFS (Fig. 3D) for CCI (≥ 1 vs. 0). (C, *p* < 0.001) and RFS (D, *p* < 0.001)

Furthermore, the impact of comorbidities according to the CCI was examined in uni- and multivariate survival analysis. In this regard, advanced patients’ age showed a highly positive correlation with prevalence of comorbidities according to CCI (Fig. 3A). There was no difference between male/female distribution (Fig. 3B). Univariate survival analysis revealed a significantly reduced survival for a CCI ≥ 1, with a five-year OAS for the entire cohort of 84.6% (CCI = 0) and 65.1% (CCI ≥ 1) (HR = 3.275, CI = 2.080–5.156, *p* <  0.001) (Fig. 3C).

For RFS five-year survival was 78.2% (CCI = 0) and 58.1% (CCI ≥ 1) (Fig. 3D, HR = 2.505, 95% CI = 1.685–3.725, *p* <  0.001). In contrast to univariate analysis, no significant correlation between impaired survival and elevated comorbidities was found after applying multivariate Cox regression (HR = 1.291, 95% CI = 0.821–2.030, *p* = 0.269) (Tab. 2).

**Fig. 4:**
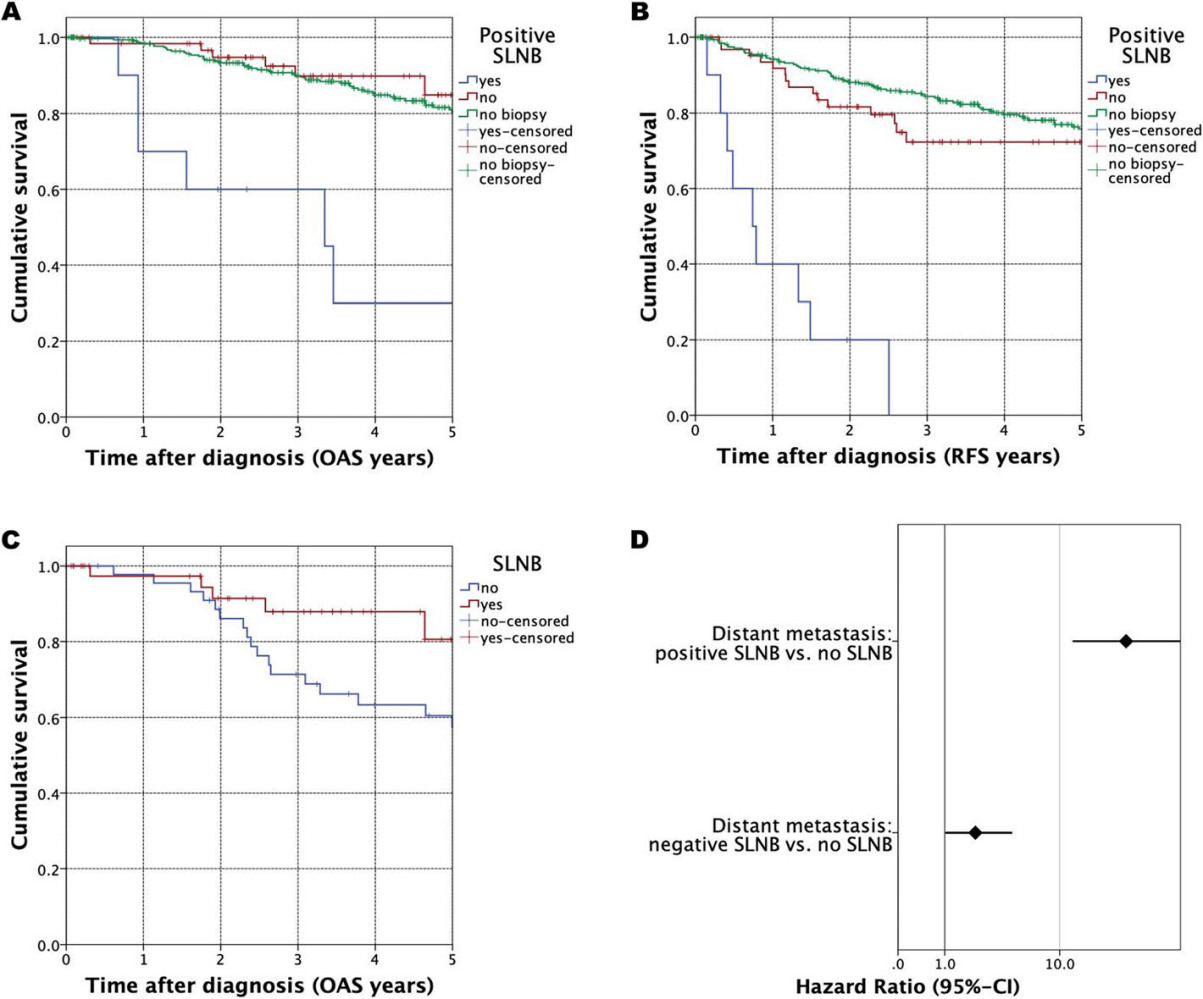
Survival in HNM patients: Kaplan-Meier curves for OAS (Fig. 4A) and RFS (Fig. 4B) differentiated by accomplished SLNB as well as result of SLNB. A: (HR = 6.386, CI = 2.727–14.956, *p* < 0.001), B: (HR = 14.442, CI = 6.974–29.906, *p* < 0.001). Figure 4C: Kaplan-Meier curve for OAS in UICC stage II patients, differentiated whether SLNB was applied or not (HR = 0.360, CI = 0.134–0.962, *p* = 0.042); Fig. 4D: Forest plots for SLNB as a predictor for distant metastasis in HNM (pos. SLNB vs. no SLNB: HR = 28.458, CI = 12.463–64.978, *p* < 0.001) (neg. SLNB vs. no SLNB: HR = 2.149, CI = 1.041–4.433, *p* = 0.038)

As a main aspect of this analysis, the significance of SLNB on outcome of HNM patients was examined. In the present retrospective study, 79 patients received treatment with SLNB. Up to nine SLNs were evaluated, in the majority of cases (63.2%), just one or two SLNs were excised. Pathohistologically verified positive SLNs were detected in 10 patients, in eight cases 1 positive node was detected, in two patients two SLNs were positive. 90% of patients with positive SLNs received a completing neck dissection. In univariate survival analysis a positive SLNB was significantly correlated with impaired OAS of the entire cohort when compared to patients without node biopsy (HR = 6.386, 95% CI = 2.727–14.956, *p* <  0.001) (Fig. 4A). For RFS, the harmful impact of histopathologically verified lymph node metastasis by SLNB could, similar to OAS, be substantiated in univariate analysis (HR = 14.442, 95% CI = 6.974–29.906, *p* <  0.001) (Fig. 4B, Tab. 2). When using multivariate Cox regression, this observation was substantiated (HR 0.046, 95% CI = 1.779–9.200, *p* = 0.001) (Tab. 2). Furthermore, we conducted subgroup analysis in UICC II patients questioning whether the sole implementation of SLNB might be correlated with improved outcome of HNM patients. As a result, a significantly positive effect of utilizing SLNB in advanced tumor stages was found (HR = 0.360, 95% CI = 0.134–0.962, *p* = 0.042) (Fig. 4C). Additionally, the impact of a verified lymph node metastasis by SLNB on occurrence of distant metastasis in the further course of disease was evaluated. In this regard, a positive SLNB significantly increased patients’ chance to develop distant metastasis in the further course of disease (HR = 28.458, CI = 12.463–64.978, *p* <  0.001) (Fig. 4D).

**Fig. 5:**
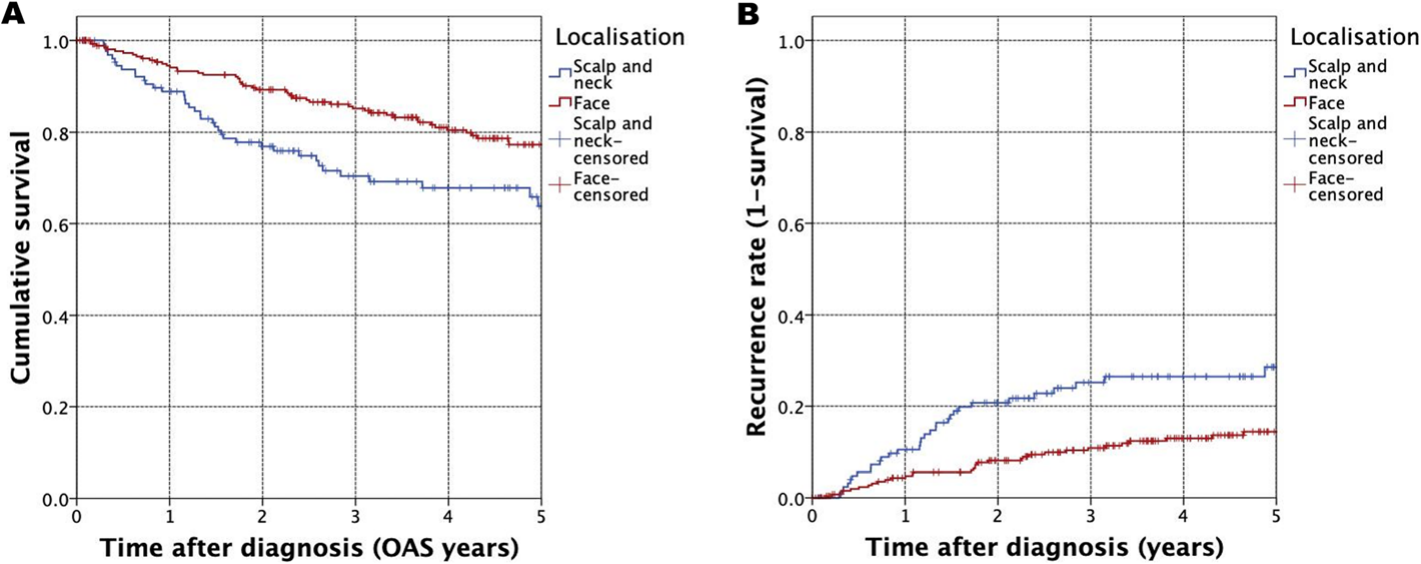
Kaplan-Meier curve for OAS (Fig. 5A) and cumulative recurrence rate (Fig. 5B) for HNM localisation face vs. scalp and neck (A: *p* = 0.021) (B: *p* < 0.001)

Our results indicate a differential oncological outcome based on distinct tumor localisations. HNM located in the face were significantly associated with impaired OAS and RFS, compared to scalp and neck melanoma (HR = 0.625, 95% CI = 0.419–0.931, *p* = 0.021, Fig. 5A) (HR = 2.482, CI = 1.524–4.042, *p* <  0.001, Fig. 5B). This result could even be more substantiated by adjusting for covariates using multivariate Cox regression (HR = 0.498, 95% CI = 0.309–0.801, *p* = 0.004) (Tab. 2).

Furthermore, age at diagnosis, histologic subtype, and resection margin proved to be significant independent risk factors for RFS (Tab. 2). Relating thereto, surgical margins ≥5 mm were clearly associated with improved RFS (HR = 0.532, CI = 0.317–0.893, *p* = 0.017). Resection margins ≥10 mm led to further beneficial aspects in RFS of HNM patients which was confirmed by multivariate analysis (HR = 0.255, CI = 0.145–0.450, *p* <  0.001) (Tab. 2).

When differentiating distinct histological subtypes, nodular melanoma of the head and neck occured, in contrast to lentigo maligna melanoma, as the most frequent histological subtype in our study. In view of survival outcome of HNM patients, nodular melanoma could be identified to clearly impair RFS (HR = 4.181, CI = 2.220–7.876, *p* <  0.001) (Tab. 2).

## Discussion

In previous studies, authors have shown unfavorable prognosis of HNM compared to melanoma of other tumor sites [15, 16]. However, our results did not support this finding. In a comprehensive review of cutaneous malignant melanoma in an European collective, stage II patients displayed a five year RFS of 56% and an OAS of 41–71% whereas similar results were seen in our population-based HNM cohort (Fig. 2A/B) [17]. Especially for stage I tumors, a diverging gap in survival could be seen between stage IA and stage IB tumors (Fig. 2A/B). With a five-year RFS of 71.7%, this explicit group of HNM patients represents an often underestimated subgroup of early melanoma, even though the risk for disease recurrence needs a more comprehensive therapy [18].

The relationship between increased prevalence of comorbidities and deleterious outcome in cancer patients has been analyzed in various tumor entities [19, 20]. For cutaneous malignant melanoma, a comprehensive cancer registry-based study on a Danish population revealed more advanced tumor stages to be prevalent in patients with increased comorbidity levels [21]. However, for HNM, no comparable analyses were published, so far. In our study, we substantiate the idea of severe comorbidities being associated with impaired outcome of cancer patients (Fig. 3C/D).

Among HNM patients, a significantly poorer prognosis is reported for distinct tumor localisations [10, 22]. Regarding this, our population-based study supports previous results of poor outcome in non-face located HNM patients (Fig. 5, Tab. 2). In this regard, uni- as well as multivariate analysis indicated decreased survival for HNM being located in the scalp or neck (Tab. 2). In an attempt to explain this observation, melanoma located in the face were reported to develop regional metastasis at a fewer extend. In this regard, facial melanoma are more accessible to clinical examination due to a better visibility and thus get resected in earlier tumor stages [10].

In our study, we were able to confirm resection margins to be a strong and independent prognostic factor determining survival in HNM. Furthermore, nodal melanoma exhibits the worst outcome among all histopathological subtypes. Concerning loco-regional metastasis, the lymphatic drainage of the head and neck region undoubtedly comprises a complex and extremely variable system [16]. Although END represents a possible approach of loco-regional tumor control, END is currently viewed as a surgical procedure of regional lymph node management which might entail only a modest benefit for selected patients [23]. Therefore, elective cervical lymphadenectomy in patients with no clinical or pathohistological signs of lymph node involvement is more and more critically seen in HNM [14]. Additionally, the already characterized variability of the cervical lymphatic drainage often leads to false negative results of cervical lymphadenectomy. Accordingly, various studies observed only a faint effect on survival for HNM patients after receiving elective lymph node dissection of the head and neck [23, 24]. In our case, only 45.5% of performed SLNBs were located in regions which are addressed by a conventional END. By illuminating different aspects of END in treatment of HNM, most authors critically assessed this procedure and eventually do not recommend END as first-line therapy [18]. In contrast, for melanoma of the coronal scalp and face with signs of lymph node involvement, parotidectomy as an additional procedure is regarded as a valuable tool to improve loco-regional tumor control [25]. Hereby unsurprisingly, SLNB has evolved as state of the art procedure, however, not primarily to replace curative lymphadenectomy, but as an appropriate staging tool especially for intermediate-thickness tumors of 1–4 mm in HNM [3, 5]. Particularly when evaluating our stage I HNM patients, no prognostic benefit to SLNB (data not shown) could be attributed. In contrast, for higher tumor stages, applying SLNB significantly resulted in improved outcome of HNM patients. Relating thereto, the sole implementation of SLNB provided a survival benefit for HNM patients, beyond the broadly accepted negative prognosis, being associated with a histopathological verified lymph node metastasis after carrying out SLNB (Fig. 4C) [26].

Out of 402 HNM patients, 165 participants had an indication to perform a SLNB, hereof 79 SLNBs were carried out. 16 patients declined performing the procedure. Out of the remaining 149 patients, 79 SLNB biopsies were carried out. Hereby, it might be important to mention that the technique of a SLNB in Eastern Bavaria was only carried out / accessible at the University Hospital Regensburg at the time of the study. Additionally, several patients presented elevated comorbidities and/or a highly advanced age which would lead into a higher risk for an intubation anesthesia.

In alignment with previous studies, SLNB occurred as a safe and accurate staging procedure, accompanied by a false negative rate of 4.3% [26, 27]. With no facial palsy and no severe postoperative complication, SLNB in the head and neck was accompanied by no permanent complications. Although seen as a current standard staging procedure, SLNB is a diagnostic procedure provided only in larger centers [6]. In this regard, not only the accessibility of the technique but also the patient specific risk accompanied by a general anesthesia due to relevant comorbidities and an advanced age might account for the aspect that less patients than current guidelines would favor [14] received SLNB.

For HNM patients with histopathologically verified SLN metastasis, the need for a complete lymphadenectomy was frequently questioned. In this regard, ultrasound-based nodal observation should be considered as the therapy of first choice [4, 28]. However, numerous HNM patients received surgical therapy in smaller institutions with limited resources which therefore limits accessibility of SLNB particularly for early stage tumours. Especially for intermediate and advanced stage HNMs, we highly recommend interdisciplinary treatment in specialized medical centers to guarantee “standard of care therapy”. Particularly the increased risk of distant disease recurrence in HNM patients with positive SLNB (Fig. 4D) illustrates the systemic component of this malignancy, demanding state of the art systemic treatment [29]. Despite our study entailing several minor limitations due to a low number of SLNB cases and despite the retrospective manner of conception, we were able to specifically define and address risk groups in HNM patients. Beyond this, the prognostic role of SLNB could be clearly evaluated and confirmed in this comprehensive multicenter population-based cohort study. Our data is of utmost importance, when it comes to implement new staging strategies with a strong prognostic relevance. Furthermore, our study aims at early defining risk groups among HNM patients and by intensifying adjuvant treatment at an early stage of disease, helping to prolong survival in this complex and quite particular tumor entity.

## Conclusions

For the entity of HNM, our study defines risk factors and prognostic markers in outcome of HNM patients. As a result, the effect of distinct histopathologic subtypes and resection margins could be confirmed in this manner. Additionally, our data underline the significance of distinct tumor localisations for regional metastasis in postoperative follow-up of HNM.

As a main aspect of this study, we were able to point out the profound prognostic impact of SLNB on patients’ outcome in HNM. In this regard, not only a positive SLN was significantly linked to a dismal prognosis, the sheer application of SLNB provided an ameliorated outcome of cancer patients. Taken together we recommend performing SLNB in HNM, particularly for higher tumor stages as an effective staging tool, helping to indicate the need for adjuvant treatment modalities.

## Data Availability

The datasets used and/or analyzed during the current study are available from the corresponding author on reasonable request.

## Abbreviations

AJCC: American Joint Committee on Cancer
CCI: Charlson comorbidity index
CI: Confidence interval
END: Elective neck dissection
HR: Hazard ratio
HNM: Head and neck melanoma
OAS: Overall survival
RFS: Recurrence free survival
SLNB: Sentinel lymph node biopsy
STARD: Standards for Reporting of Diagnostic Accuracy Studies
UICC: Union for International Cancer Control
